# Cyclophilin A Inhibitors Suppress Proliferation and Induce Apoptosis of MKN45 Gastric Cancer Stem-like Cells by Regulating CypA/CD147-Mediated Signaling Pathway

**DOI:** 10.3390/ijms24054734

**Published:** 2023-03-01

**Authors:** Hee Jeong Cho, Hye Jin Jung

**Affiliations:** 1 Department of Life Science and Biochemical Engineering, Graduate School, Sun Moon University, Asan 31460, Republic of Korea; 2 Department of Pharmaceutical Engineering and Biotechnology, Sun Moon University, Asan 31460, Republic of Korea; 3 Genome-Based BioIT Convergence Institute, Sun Moon University, Asan 31460, Republic of Korea

**Keywords:** gastric cancer stem cells, cyclophilin A, CD147, compound 9, cyclosporin A

## Abstract

Gastric cancer stem cells (GCSCs) are a subgroup of gastric cancer (GC) cells with high self-renewal and multi-lineage differentiation abilities that lead to tumor initiation, metastasis, drug resistance, and tumor relapse. Therefore, the eradication of GCSCs can contribute to the effective treatment of advanced or metastatic GC. In our previous study, compound 9 (C9), a novel derivative of nargenicin A1, was identified as a potential natural anticancer agent that specifically targeted cyclophilin A (CypA). However, its therapeutic effect and molecular mechanisms of action on GCSC growth have not been assessed. In this study, we investigated the effects of natural CypA inhibitors, including C9 and cyclosporin A (CsA), on the growth of MKN45-derived GCSCs. Compound 9 and CsA effectively suppressed cell proliferation by inducing cell cycle arrest at the G0/G1 phase and promoted apoptosis by activating the caspase cascade in MKN45 GCSCs. In addition, C9 and CsA potently inhibited tumor growth in the MKN45 GCSC-grafted chick embryo chorioallantoic membrane (CAM) model. Furthermore, the two compounds significantly decreased the protein expression of key GCSC markers including CD133, CD44, integrin α6, Sox2, Oct4, and Nanog. Notably, the anticancer activities of C9 and CsA in MKN45 GCSCs were associated with the regulation of CypA/CD147-mediated AKT and mitogen-activated protein kinase (MAPK) signaling pathways. Collectively, our findings suggest that the natural CypA inhibitors C9 and CsA could be novel anticancer agents used to combat GCSCs by targeting the CypA/CD147 axis.

## 1. Introduction

Gastric cancer (GC) is the fifth most commonly diagnosed cancer and the third leading cause of cancer-related mortality worldwide [1]. The main causes of GC include *Helicobacter pylori* infection, E-cadherin gene mutations, interleukin gene polymorphisms, and poor dietary habits [2]. In early stages of GC, the use of adjuvant radiation therapy and chemotherapy after surgical resection significantly reduces the risk of recurrence and improves overall survival [3]. However, despite recent improvements in treatment options, the prognosis of patients with advanced or metastatic GC remains poor, with a median overall survival of 10–12 months [4]. Accumulating evidence indicates that gastric cancer stem cells (GCSCs) contribute to refractory features of GC, such as metastasis, recurrence, heterogeneity, resistance to radiotherapy and chemotherapy, and immune evasion [5,6]. Gastric cancer stem cells are a small subpopulation within tumors with self-renewal, differentiation, and tumor-initiating abilities [5,6]. Although several possible targets were discovered (cell surface markers, microenvironmental factors, and signaling pathways) that may regulate the biological characteristics of GCSCs, exploring potential new therapeutic strategies to combat GCSCs can significantly improve the clinical outcome of GC treatment [5,6].

Cyclophilin A (CypA) belongs to the immunophilin family and catalyzes the isomerization of peptidyl–prolyl bonds [7]. It is a key molecule that regulates many biological functions, including molecular chaperoning, protein folding, protein transport, immune regulation, and cellular signaling [7]. It is also a cytoplasmic protein that is secreted from cells in response to proinflammatory stimuli [8]. Secreted CypA binds to CD147, a glycosylated cell surface transmembrane protein of the immunoglobulin superfamily that mediates intercellular communication and intracellular responses [9]. Recent studies have demonstrated that activation of the CypA/CD147 axis induces proliferation, anti-apoptosis, metastasis, and drug resistance in cancer cells, and it increases the survival of cancer stem cells (CSCs) [10]. Furthermore, CypA promotes self-renewal, proliferation, and radiotherapy resistance in glioma stem cells by upregulating Wnt/β-catenin signaling [10,11]. CD147 enhances the release of small extracellular vesicles during the differentiation of colon CSCs, which can increase the invasive potential of colon cancer cells [10,12]. Inhibition of CD147 increases chemosensitivity by suppressing CSC subpopulations in colon and pancreatic cancer cells [13,14]. CypA/CD147 activation is essential for maintaining CSC features in breast cancer cells through signal transducer and activator of transcription 3 (STAT3) signaling, such as their tumorsphere-forming ability, the expression of stemness markers, and chemoresistance [10,15]. Therefore, the CypA/CD147 axis may be a potential therapeutic target for CSC eradication.

**Figure 1:**
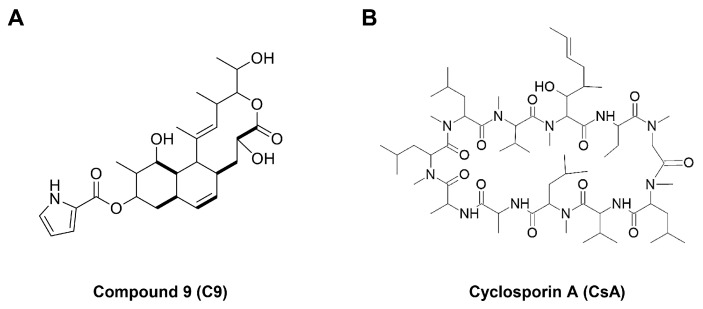
The chemical structures of (**A**) C9 and (**B**) CsA.

Recently, 23-demethyl 8,13-deoxynargenicin (C9) was discovered as a new anticancer agent that targets CypA/CD147 interaction [16] (Figure 1A). Unlike its parent compound with effective antibacterial activity, nargenicin A1, C9 possesses potential anticancer activity against various cancer cells, including GC [17]. C9 not only inhibits the expression of CypA and CD147 but also binds to CypA and downregulates CD147-mediated mitogen-activated protein kinase (MAPK) signaling pathways in GC cells, including c-Jun N-terminal kinase (JNK) and extracellular signal-regulated protein kinase 1/2 (ERK1/2) [16]. C9 suppresses the proliferation, migration, invasion, and angiogenesis of GC cells [16,17,18]. In addition, CypA-silencing inhibited the proliferation and induced apoptosis of GC cells by blocking CD147 expression and CD147-mediated downstream signaling pathways, indicating that the CypA/CD147 interaction may play an important role in GC development [16]. Consistent with these results, cyclosporin A (CsA), a representative CypA inhibitor with a wide range of biological activities such as immunosuppressive, anti-inflammatory, antifungal, and antitumor effects, inhibited the growth of GC cells by inducing cell cycle arrest and apoptosis [10,19,20] (Figure 1B). CsA also enhances the docetaxel-induced apoptotic pathway via inhibition of nuclear factor-κB (NF-κB) activation in GC cells [21]. However, no study has shown that targeting the CypA/CD147 axis using CypA inhibitors, including C9 and CsA, can inhibit GCSC survival. Here, we investigated the anticancer effects and underlying molecular mechanisms of CypA inhibitors, including C9 and CsA, against GCSCs for the first time.

## 2. Results

### 2.1. C9 and CsA Inhibit Proliferation of MKN45-Derived GCSCs Both In Vitro and In Vivo

**Figure 2:**
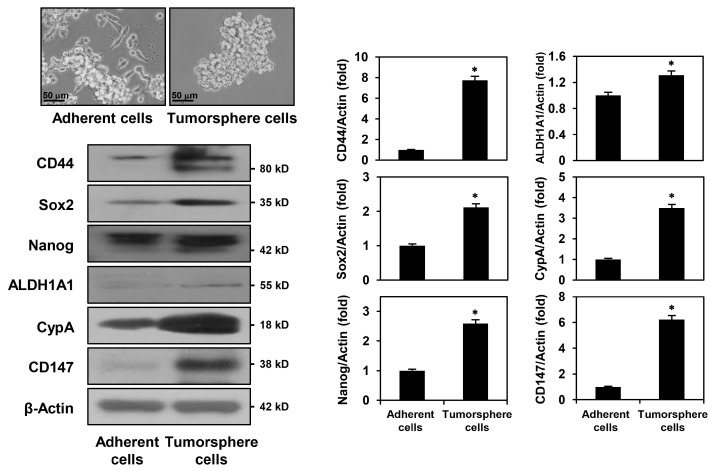
Expression levels of key gastric cancer stem cell (GCSC) markers, CypA, and CD147 in MKN45 adherent and tumorsphere cells. Protein levels were detected via Western blot analysis using specific antibodies and were further quantified using densitometry. β-actin levels were used as an internal control. The ratio of each target protein to β-actin for adherent cells was normalized to onefold. * *p* < 0.05 vs. the adherent cells.

To enrich the GCSC population from the GC cell line, MKN45 cells were grown in serum-free spheroid suspension cultures containing epidermal growth factor (EGF) and basic fibroblast growth factor (bFGF) [22]. The stem-like properties of GC cells are accompanied by the upregulation of stemness-related factors. MKN45 tumorsphere cells overexpressed key CSC markers, including CD44, Sox2, Nanog, and aldehyde dehydrogenase 1A1 (ALDH1A1), compared to those of MKN45 adherent cells cultured in 10% serum-supplemented medium (Figure 2). These data indicate that serum-free tumorsphere cultures can propagate the MKN45-derived GCSC population. In addition, the expression levels of CypA and CD147 were remarkably increased in MKN45 tumorsphere cells compared to those of MKN45 adherent cells, suggesting that the CypA/CD147 axis may play a critical role in the maintenance of GCSCs (Figure 2).

**Figure 3:**
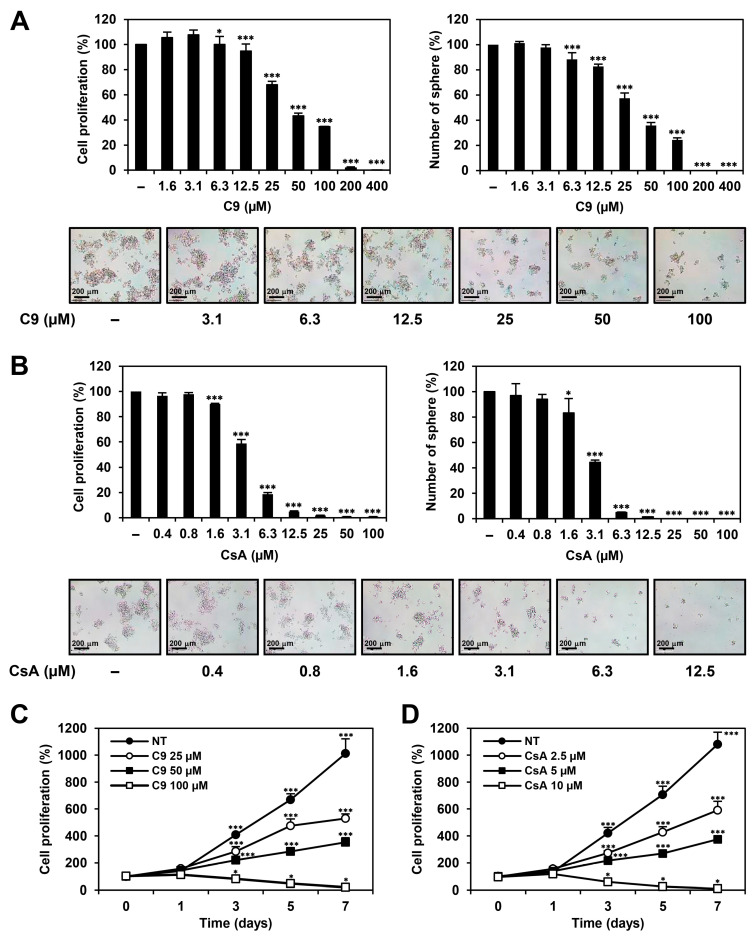
Dose- and time-dependent effects of C9 and CsA on the proliferation of MKN45 GCSCs. MKN45-derived GCSCs were treated with the indicated concentrations of (**A**,**C**) C9 and (**B**,**D**) CsA for seven days. Cell proliferation was measured using the CellTiter-Glo^®^ luminescent assay system. Formed tumorspheres were counted under an optical microscope. * *p* < 0.05, *** *p* < 0.001 vs. the control.

To investigate the effects of C9 and CsA on the proliferation of GCSCs, MKN45-derived GCSCs were treated with C9 and CsA at various concentrations for seven days. Cell proliferation was measured using an ATP-monitoring luminescence assay. Compound 9 and CsA dose-dependently inhibited the proliferation of MKN45 GCSCs with 47.1 and 3.6 μM of IC_50_ value, respectively (Figure 3A,B). Both compounds also suppressed the proliferation of MKN45 GCSCs in a time-dependent manner (Figure 3C,D). Furthermore, C9 and CsA significantly suppressed the tumorsphere-forming ability of the MKN45 GCSCs (Figure 3A,B). The effects of C9 and CsA on the self-renewal capacity of MKN45 GCSCs were further evaluated via limiting dilution assay, a widely used method to quantify stem cell frequency. The C9- and CsA-treated cells showed six- and eightfold lower spheroid forming frequencies, respectively, compared to untreated control cells, indicating that C9 and CsA effectively inhibited the self-renewal of MKN45 GCSCs (Figure S1). Taken together, these results demonstrate that CypA inhibitors suppressed the proliferation of GCSCs in a dose- and time-dependent manner. Notably, the inhibitory effects of C9 and CsA on MKN45 GCSC growth decreased after compound washout, indicating that C9 and CsA may induce reversible anticancer effects on MKN45 GCSCs (Figure S2). In addition, C9 and CsA inhibited the viability of 267B1 prostate epithelial cells, a human normal cell line, with 236.4 and 129.6 μM of IC50 value, respectively, implying that the antiproliferative activities of C9 and CsA against MKN45 GCSCs are not due to mere cytotoxicity of the compounds (Figure S3).

**Figure 4:**
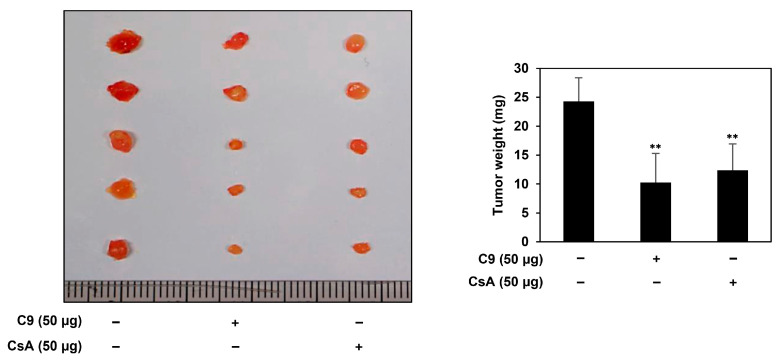
Effects of C9 and CsA on tumor growth derived by MKN45 GCSCs in a CAM model. MKN45-derived GCSCs were mixed with Matrigel in the absence or presence of the compounds (50 µg/egg) and placed onto the CAM surface of fertilized chick eggs. After incubation for seven days, the CAMs were observed, the formed tumors were retrieved, and the tumors’ weights were calculated. ** *p* < 0.005 vs. the control.

To further evaluate the effects of C9 and CsA on the tumorigenic potential of GCSCs in vivo, a modified chorioallantoic membrane (CAM) assay (injected with MKN45 GCSCs) was performed. As shown in Figure 4, the tumor weight of the control group was 24.3 mg, whereas those of the C9 and CsA treatment were 10.3 and 12.4 mg, respectively. These data indicate that the CypA inhibitors effectively inhibited GCSC-derived tumor growth in vivo. Notably, C9 exhibited a better growth-inhibitory effect on MKN45 GCSCs in the CAM tumor model than CsA, suggesting that C9 may possess superior therapeutic potential to eliminate GCSCs than CsA in vivo.

### 2.2. C9 and CsA Suppress Expression of Key Cancer Stemness Markers in MKN45-Derived GCSCs

**Figure 5:**
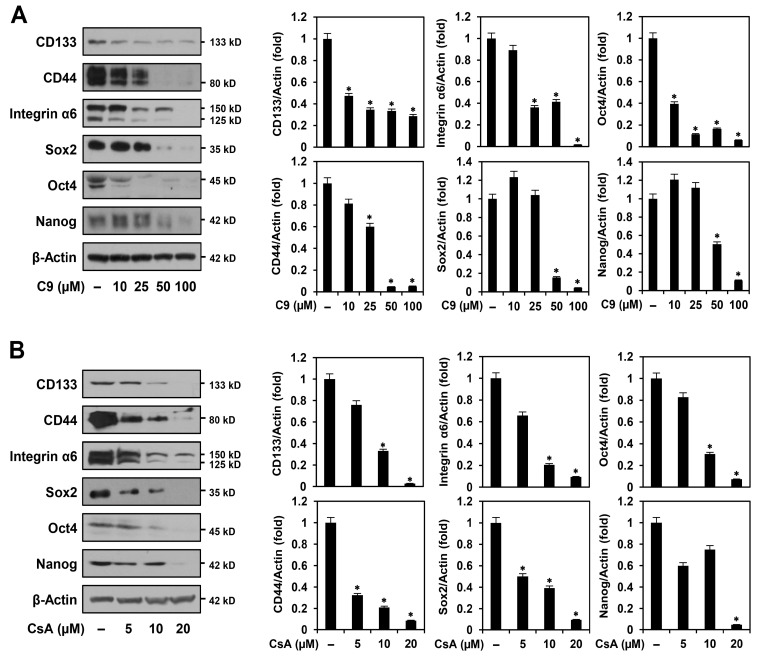
Effects of C9 and CsA on the expression of cancer stemness markers in MKN45 GCSCs. MKN45-derived GCSCs were treated with the indicated concentrations of (**A**) C9 and (**B**) CsA for 72 h. Protein levels were detected via Western blot analysis using specific antibodies and were further quantified using densitometry. β-actin levels were used as an internal control. The ratio of each target protein to β-actin for untreated control was normalized to onefold. * *p* < 0.05 vs. the control.

Several stem cell regulators were identified as key biomarkers for GCSC maintenance [6,22]. Cell surface proteins, such as CD133, CD44, and integrin α6, are associated with higher tumorigenic and metastatic potential in GCSCs [22,23]. Transcription factors, including Sox2, Oct4, and Nanog, play important roles in maintaining the pluripotency and self-renewal of GCSCs [22,24]. Therefore, the inhibition of these cancer stemness regulators is necessary to effectively eradicate GCSCs. We investigated whether C9 and CsA affected the expression of GCSC biomarkers. As shown in Figure 5, the two compounds significantly decreased the expression levels of CD133, CD44, integrin α6, Sox2, Oct4, and Nanog in MKN45 GCSCs. These results indicate that the anticancer effects of C9 and CsA on GCSCs are related to the downregulation of major GCSC biomarkers.

### 2.3. C9 and CsA Induce Cell Cycle Arrest and Apoptosis in MKN45-Derived GCSCs

**Figure 6:**
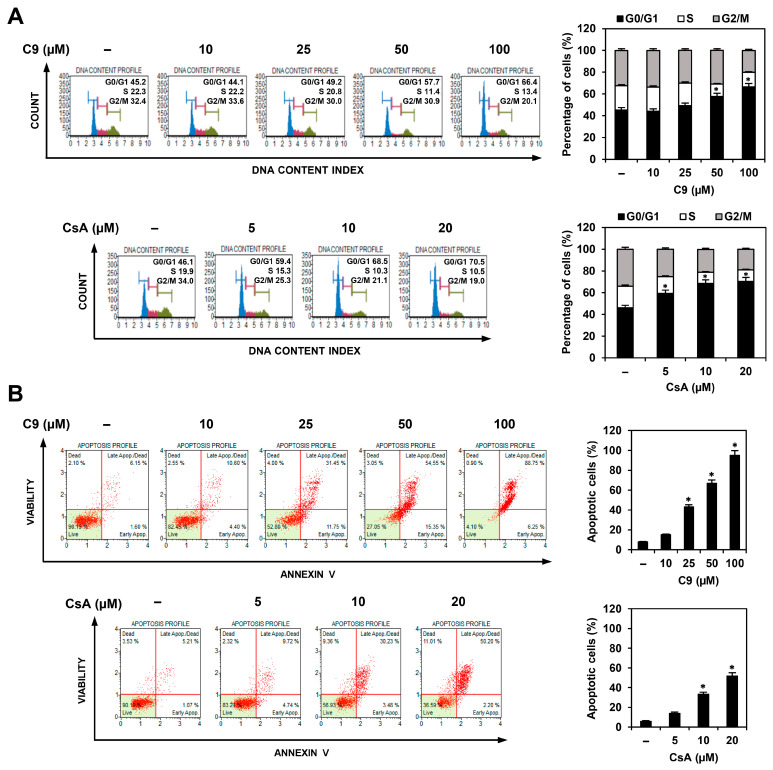
Effects of C9 and CsA on the cell cycle and apoptosis in MKN45 GCSCs. (**A**,**B**) MKN45-derived GCSCs were treated with the indicated concentrations of C9 and CsA for 72 h. (**A**) Effects of C9 and CsA on the cell cycle. Cell cycle distribution was detected using a Muse Cell Analyzer with a Muse^®^ Cell Cycle kit. (**B**) Effects of C9 and CsA on apoptotic cell death. Apoptotic cells were detected using a Muse Cell Analyzer with a Muse^®^ Annexin V & Dead Cell kit. * *p* < 0.05 vs. the control.

To assess whether C9 and CsA inhibited the proliferation of MKN45 GCSCs by regulating the cell cycle and apoptosis, we first investigated the effects of C9 and CsA on the cell cycle distribution of MKN45 GCSCs using flow cytometry. Compared with the untreated control cells, both compounds significantly increased the cell population in the G0/G1 phase and decreased the cell population in the S and G2/M phases (Figure 6A). These results indicated that C9 and CsA induced G0/G1 cell cycle arrest in MKN45 GCSCs. Next, the effects of C9 and CsA on the apoptosis of MKN45 GCSCs were analyzed using flow cytometry. Both compounds increased the proportion of apoptotic cells in a dose-dependent manner (Figure 6B).

**Figure 7:**
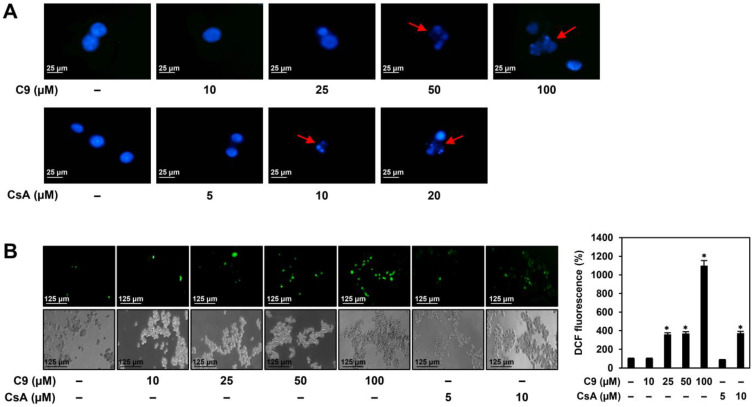

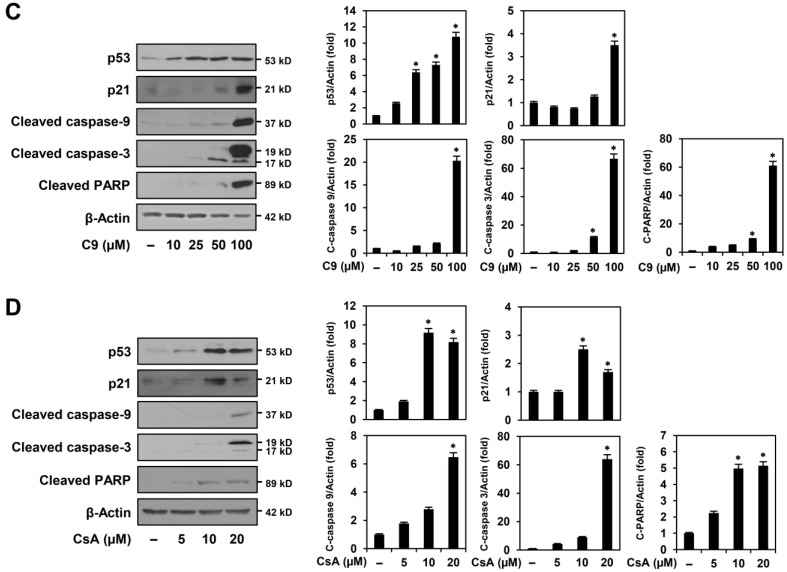
Effects of C9 and CsA on the apoptotic characteristics of MKN45 GCSCs. (**A**) Effects of C9 and CsA on nuclear morphology. MKN45-derived GCSCs were treated with the indicated concentrations of each compound for 72 h. Changes in nuclear morphology were monitored via DAPI staining under a fluorescence microscope. The condensed and fragmented nuclei are indicated by red arrows. (**B**) Effects of C9 and CsA on intracellular ROS generation. MKN45-derived GCSCs were treated with the indicated concentrations of each compound for 6 h. ROS levels were detected with H_2_DCFDA using a fluorescence microscope and were further quantified via densitometry. The level of DCF fluorescence for untreated control was normalized to 100%. (**C**,**D**) Effects of C9 and CsA on the expression of apoptosis regulators. MKN45-derived GCSCs were treated with the indicated concentrations of each compound for 72 h. Protein levels were detected via Western blot analysis using specific antibodies and were further quantified using densitometry. β-actin levels were used as an internal control. The ratio of each target protein to β-actin for untreated control was normalized to onefold. * *p* < 0.05 vs. the control.

To further elucidate the apoptosis-promoting effects of C9 and CsA in MKN45 GCSCs, we examined whether they affected several representative features of apoptosis. As shown in Figure 7A, C9 and CsA caused nuclear condensation and fragmentation of MKN45 GCSCs, which are the morphological hallmarks of apoptosis [25]. The accumulation of intracellular reactive oxygen species (ROS) is closely related to the activation of apoptosis [26]. As shown in Figure 7B, C9 and CsA increased ROS generation in MKN45 GCSCs in a dose-dependent manner. The tumor suppressor p53 acts as an upstream regulator of the caspase-mediated apoptotic pathway [25]. Moreover, p53 transcriptionally activates p21, an inhibitor of cyclin-dependent kinases that are required for cell cycle progression. Caspases are essential effectors of apoptosis that are activated by proteolytic cleavage and promote cell apoptosis by cleaving a set of vital proteins, including poly (ADP-ribose) polymerase (PARP) [25,27]. As shown in Figure 7C,D, C9 and CsA upregulated the protein levels of p53, p21, cleaved caspase-9, caspase-3, and PARP in MKN45 GCSCs. Taken together, these results demonstrate that the antiproliferative effects of CypA inhibitors on MKN45 GCSCs resulted from the induction of G0/G1 cell cycle arrest and apoptosis via the activation of the ROS-mediated caspase cascade.

### 2.4. C9 and CsA Affect CypA/CD147-Mediated Signaling Pathways in MKN45-Derived GCSCs

**Figure 8:**
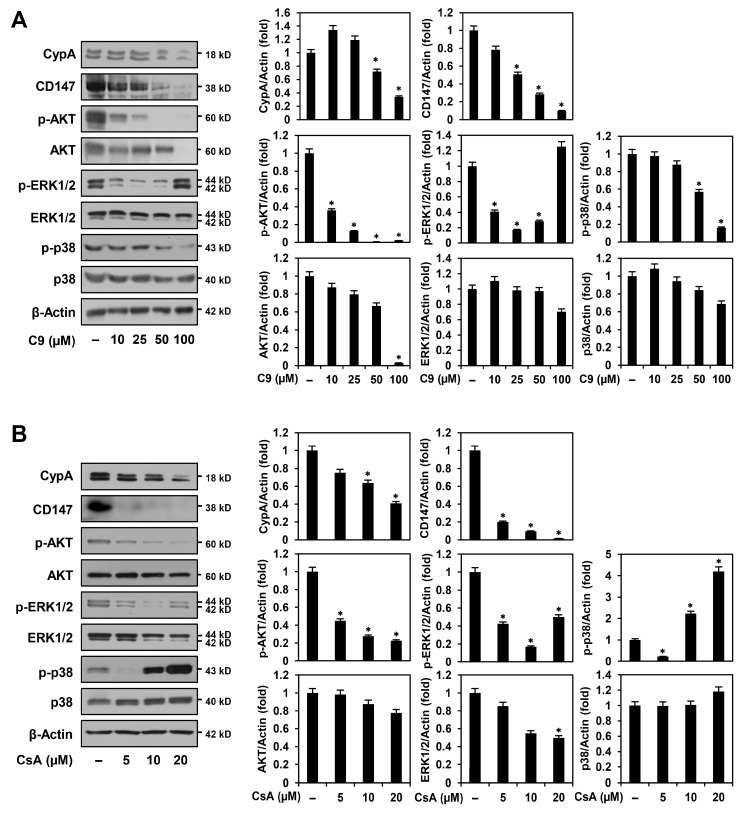
Effects of C9 and CsA on the CypA/CD147-mediated signaling pathways in MKN45 GCSCs. (**A**,**B**) MKN45-derived GCSCs were treated with the indicated concentrations of each compound for 72 h. Protein levels were detected via Western blot analysis using specific antibodies and were further quantified using densitometry. β-actin levels were used as an internal control. The ratio of each target protein to β-actin for untreated control was normalized to onefold. * *p* < 0.05 vs. the control.

Cyclophilin A and CD147 are overexpressed in various types of cancers, and CypA/CD147 interaction can activate multiple oncogenic signaling pathways, including phosphatidylinositol-3-kinase (PI3K)/AKT and MAPKs, thereby promoting the proliferation, anti-apoptosis, metastasis, angiogenesis, drug resistance, and stemness of cancer cells [10,16,28]. To determine whether the anticancer activities of C9 and CsA against MKN45 GCSCs were associated with downregulation of the CypA/CD147 axis, we first investigated the effects of C9 and CsA on CypA and CD147 expression. As shown in Figure 8A,B, the expressions of CypA and CD147 were reduced by treatment with C9 and CsA in a dose-dependent manner in MKN45 GCSCs. Next, we examined the effects of C9 and CsA on the activation of CypA/CD147-mediated downstream signaling effectors including AKT, ERK1/2, and p38. The results showed that C9 and CsA more effectively suppressed the expression levels of phosphorylated forms than unphosphorylated AKT, ERK1/2, and p38 proteins in MKN45 GCSCs (Figure 8A,B). However, the phosphorylation of ERK1/2 and p38 MAPKs showed a biphasic response dependent on the concentrations of C9 and CsA. The activation of MAPKs is widely associated with anti-apoptotic functions but can induce pro-apoptotic mechanisms in a cell context-specific and cell type-specific manner [29]. Therefore, our data imply that C9 and CsA may cause apoptosis of MKN45 GCSCs by simultaneously regulating the dual role of MAPKs based on their treatment concentrations. Collectively, these results suggest that the anticancer effects of CypA inhibitors on GCSCs involve the regulation of major CypA/CD147-mediated oncogenic signaling pathways, such as AKT, ERK1/2, and p38, by inhibiting the expressions of CypA and CD147.

## 3. Discussion

Despite surgical treatment, radiotherapy, and chemotherapy, GC remains a malignancy with a high mortality rate [1,3,4]. Gastric cancer stem cells are a subgroup of GC cells with high self-renewal and multi-lineage differentiation abilities, leading to tumor initiation, metastasis, drug resistance, and tumor recurrence [22,30,31]. Therefore, targeting GCSCs is considered a powerful therapeutic strategy to improve treatment outcomes in patients with advanced or metastatic GC [31]. Although several GCSC-targeted therapies that modulate CSC-related signaling pathways, the CSC microenvironment, and CSC biomarkers were developed, further exploration of potential therapeutic targets and drugs that can effectively inhibit GCSC proliferation and promote GCSC apoptosis may contribute to overcoming treatment resistance and GC recurrence [5].

Accumulating evidence revealed that the CypA/CD147 axis plays an important role in the initiation, growth, and survival of CSCs [9,10,11,15]. CypA/CD147 activation induces CSC features in breast cancer cells through the STAT3 and solute carrier family 34 member 2 (SLC34A2)/phosphatidylinositol-3-kinase (PI3K)/AKT/SRY-box transcription factor 2 (SOX2) signaling pathways [15,32]. CypA also promotes self-renewal, proliferation, and radiotherapy resistance in glioma stem cells by upregulating Wnt/β-catenin signaling [11]. In addition, the gene encoding CypA is one of the most stably expressed essential genes in the CSC phenotype, and peptidyl–prolyl isomerase activity increases the sphere formation, self-renewal, and metastasis of CSCs through Notch signaling [10,33]. Furthermore, CD147 induced colon cancer invasion by regulating the differentiation of colon CSCs [12]. The RNA interference-mediated gene silencing of CD147 also suppresses the proliferation and invasion of colon CSCs and enhances chemosensitivity by inhibiting stemness markers [14]. In addition, the anti-CD147 drug metuximab sensitizes pancreatic cancer cells to chemoradiotherapy by reducing the CSC subpopulation via blockade of CD44/STAT3 signaling [13]. Therefore, the CypA/CD147 axis may be a potential target for CSC elimination.

The overexpression of CypA and CD147 promotes proliferation, anti-apoptosis, invasion, migration, and angiogenesis of GC cells and is associated with poor prognosis in patients with GC [10,16,34]. In addition, the expression of CypA and CD147 was positively correlated with GC, indicating that CypA/CD147 interaction plays a crucial role in GC development [10]. In our previous studies, C9 was identified as a promising anticancer agent that targets CypA/CD147 interaction [16]. Compound 9 is a novel analog of the antibacterial macrolide nargenicin A1; however, unlike nargenicin A1, it has potential antitumor and antiangiogenic activities [16,17,18]. Notably, proteomics analysis and further functional studies demonstrated that C9 binds to CypA and downregulates the CD147-mediated MAPK signaling pathway, including JNK and ERK1/2, by inhibiting CypA and CD147 expression in GC cells [16]. Compound 9 responds by suppressing the proliferation, migration, invasion, and angiogenesis of GC cells [16,17,18]. However, whether targeting the CypA/CD147 axis by C9 affects GCSC survival has not been assessed. In the current study, we investigated the anticancer effects and underlying molecular mechanisms of CypA inhibitors, including C9 and CsA, against GCSCs for the first time.

Our results demonstrated that C9 and CsA inhibited the proliferation and promoted the apoptosis of MKN45-derived GCSCs by modulating the CypA/CD147-mediated signaling pathway. Cyclophilin A inhibitors significantly suppressed the proliferation of MKN45 GCSCs by inducing G0/G1 cell cycle arrest, leading to apoptotic cell death by activating the ROS-mediated caspase cascade. Furthermore, CypA inhibitors effectively inhibited MKN45 GCSC-derived tumor growth in an in vivo model of tumorigenesis. Notably, C9 exhibited a better growth-inhibitory effect on MKN45 GCSCs in vivo than that of CsA, suggesting that C9 may have superior therapeutic potential in eradicating GCSCs than CsA in vivo. Furthermore, the anticancer effects of C9 and CsA on MKN45 GCSCs were associated with a remarkable reduction in the expression of key GCSC markers including CD133, CD44, integrin α6, Sox2, Oct4, and Nanog. The two compounds also affected CypA/CD147-mediated oncogenic signaling pathways, including AKT, ERK1/2, and p38, by inhibiting the expression of CypA and CD147 in MKN45 GCSCs. However, ERK1/2 and p38 MAPKs exhibited concentration-dependent biphasic responses to C9 and CsA, implying that treatment with high doses of these compounds may simultaneously modulate CypA and other unidentified molecular targets. Taken together, our findings suggest that the CypA/CD147 axis is a promising therapeutic target to combat GCSCs, and that the CypA inhibitors C9 and CsA can be utilized as novel anticancer agents targeting GCSCs in monotherapy or in combination therapy with approved drugs. Further molecular mechanisms and in vivo animal model studies are required to more comprehensively evaluate the efficacy and safety of CypA inhibitors as potential therapeutic agents for overcoming the chemoresistance and recurrence of GC.

## 4. Materials and Methods

### 4.1. Materials

Compound 9 was isolated from the culture extract of *Nocardia* sp. CS682 mutant as previously described [17]. Cyclosporin A was purchased from Sigma-Aldrich (St. Louis, MO, USA). C9 and CsA were dissolved in dimethyl sulfoxide (DMSO) at a concentration of 50 mM. RPMI-1640, fetal bovine serum (FBS), and penicillin-streptomycin-amphotericin B were purchased from Corning Cellgro (Manassas, VA, USA), R&D Systems (Minneapolis, MN, USA), and Lonza (Walkersville, MD, USA), respectively. DMEM/F12 and trypsin were obtained from HyClone (Marlborough, MA, USA). B-27 serum-free supplement, L-glutamine, and penicillin/streptomycin were purchased from Gibco (Grand Island, NY, USA). Epidermal growth factor (EGF) and basic fibroblast growth factor (bFGF) were obtained from Prospecbio (East Brunswick, NJ, USA). Accutase and Matrigel were purchased from EMD Millipore (Temecula, CA, USA) and Corning (Tewksbury, MA, USA), respectively. Heparin, DAPI, and DCFH-DA were purchased from Sigma-Aldrich (St. Louis, MO, USA). The CellTiter-Glo^®^ 2.0 Cell Viability Assay kit was purchased from Promega (Madison, WI, USA). Muse^®^ Cell Cycle and Muse^®^ Annexin V & Dead Cell kits were purchased from Luminex (Austin, TX, USA). Antibodies against CypA (#2175), CD147 (#13287), CD44 (#37259), CD133 (#64326), integrin α6 (#3750), Sox2 (#3579), Nanog (#3580), Oct4 (#2750), ALDH1A1 (#12035), p53 (#2524), p21 Waf1/Cip1 (#2947), cleaved caspase-9 (#9501), cleaved caspase-3 (#9661), cleaved PARP (#9542), phospho-AKT (#4060), AKT (#9272), phospho-ERK1/2 (#9101), ERK1/2 (#9102), phospho-p38 (#4511), p38 (#8690), rabbit IgG (#7074), and mouse IgG (#7076) were purchased from Cell Signaling Technology (Danvers, MA, USA). The anti-β-actin (#ab6276) antibody was purchased from Abcam (Cambridge, UK).

### 4.2. Cell Culture

The human gastric adenocarcinoma cell line MKN45 was obtained from the Korean Cell Line Bank (Seoul, Republic of Korea). MKN45 adherent cells were cultured in RPMI-1640 medium supplemented with 10% FBS and 1% penicillin-streptomycin-amphotericin B. MKN45 tumorsphere cells were grown in DMEM/F12 containing 1× B-27, 5 µg/mL heparin, 2 mM L-glutamine, 20 ng/mL EGF, 20 ng/mL bFGF, and 1% penicillin/streptomycin as previously described [22]. Tumorspheres grown in serum-free media were subcultured through dissociation with Accutase. In this study, we used MKN45 tumorsphere cells at passage 2. The adherent and tumorsphere cells were maintained at 37 °C in a humidified CO_2_ incubator with 5% CO_2_ (Thermo Scientific, Vantaa, Finland).

### 4.3. Cell Proliferation Assay

Cell proliferation was measured using the CellTiter-Glo^®^ 2.0 Cell Viability Assay as previously described [22,35]. MKN45-derived GCSCs (3 × 10^3^ cells/well) were seeded in a 96-white-well culture plate using serum-free media and treated with different concentrations of each compound for seven days. Luminescence was detected using a multimode microplate reader (BioTek Inc., Winooski, VT, USA). The IC_50_ values obtained from the data were analyzed using GraphPad Prism 6 (GraphPad Software, La Jolla, CA, USA).

### 4.4. Tumorsphere Formation Assay

To evaluate the ability of a single GCSC to grow into a tumorsphere, MKN45-derived GCSCs (3 × 10^3^ cells/well) were seeded in a 96-well culture plate using serum-free media and treated with different concentrations of each compound for seven days. Tumorspheres > 70 μm in diameter were counted using an optical microscope (Olympus, Tokyo, Japan).

### 4.5. Chick Embryo Chorioallantoic Membrane (CAM) Assay

To investigate the effects of the compounds on GC tumorigenesis in vivo, a modified CAM assay was performed as previously described [22,35]. Briefly, fertilized chicken eggs were incubated at 37 °C in a humidified egg incubator for six days, and the eggshell membrane was carefully peeled away. MKN45-derived GCSCs (1 × 10^6^ cells/egg) were mixed with Matrigel (40 μL/egg) in the absence or presence of the compounds (50 μg/egg) and placed on the CAM. After incubation for seven days, the tumor formed on the CAM was retrieved, and the tumor’s weight was measured.

### 4.6. Cell Cycle Analysis

MKN45-derived GCSCs (9 × 10^4^ cells/well) were seeded in a 60-mm culture plate using serum-free media and treated with different concentrations of each compound for 72 h. The cells were harvested, fixed with 70% ethanol, and stained with 200 μL Muse^®^ Cell Cycle reagent as previously described [22]. Cell cycle distribution was analyzed using a Guava^®^ Muse^®^ Cell Analyzer (MuseSoft_V1.8.0.3; Luminex Corporation, Austin, TX, USA).

### 4.7. Apoptosis Analysis

MKN45-derived GCSCs (9 × 10^4^ cells/well) were seeded in a 60-mm culture plate using serum-free media and treated with different concentrations of each compound for 72 h. The cells were collected and stained with 100 μL Muse^®^ Annexin V & Dead Cell reagent as previously described [22,35]. Cellular apoptosis was quantitatively analyzed using a Guava^®^ Muse^®^ Cell Analyzer (MuseSoft_V1.8.0.3; Luminex Corporation, Austin, TX, USA).

### 4.8. DAPI Staining

MKN45-derived GCSCs (2 × 10^5^ cells/well) were seeded in a 24-well culture plate using serum-free media and treated with the indicated concentrations of each compound for 72 h. The cells were stained with 20 μg/mL DAPI for 30 min. The nuclear morphology of the cells was observed under a fluorescence microscope (Optinity KI-2000F, Korea Lab Tech, Seong Nam, Republic of Korea).

### 4.9. ROS Measurement

MKN45-derived GCSCs (3 × 10^5^ cells/well) were seeded in a 24-well culture plate using serum-free media and treated with the indicated concentrations of each compound for 6 h. The cells were then stained with 20 μM H_2_DCFDA for 20 min. The levels of intracellular ROS were observed under a fluorescence microscope (Optinity KI-2000F, Korea Lab Tech, Seong Nam, Republic of Korea) and quantified by measuring DCF fluorescence intensity using ImageJ 1.5 software (NIH, Bethesda, MD, USA).

### 4.10. Western Blot Analysis

Cell lysates were separated via 7.5–15% sodium dodecyl sulfate-polyacrylamide gel electrophoresis. The separated proteins were transferred onto polyvinylidene difluoride membranes (EMD Millipore, Hayward, CA, USA). The blots were blocked with 5% skim milk in Tris-buffered saline with 1 × Tween-20 (TBST) at room temperature for 1 h and then immunolabeled with primary antibodies (dilution 1:2000–1:10,000) overnight at 4 °C as previously described [22,35]. After washing with TBST, the membranes were incubated with horseradish peroxidase-conjugated secondary antibodies (dilution 1:3000) at room temperature for 1 h. Immunolabeling was performed using an enhanced chemiluminescence kit (Bio-Rad Laboratories, Hercules, CA, USA) according to the manufacturer’s instructions. Band density was analyzed using ImageJ 1.5 software (NIH, Bethesda, MD, USA). The expression levels were determined as the normalized ratio of each target protein to β-actin.

### 4.11. Statistical Analysis

The results are expressed as the mean ± standard deviation from at least three independent experiments. Statistical analysis was performed by using ANOVA with Tukey’s post-hoc test using SPSS software (version 9.0; SPSS Inc., Chicago, IL, USA). The level of statistical significance was set at *p* < 0.05.

## 5. Conclusions

In this study, we identified the anticancer activities of CypA inhibitors C9 and CsA against GCSCs. C9 and CsA inhibited the growth of MKN45-derived GCSCs both in vitro and in vivo by inducing cell cycle arrest at the G0/G1 phase and at caspase-dependent apoptosis. Both compounds significantly reduced the protein expression levels of key CSC markers, such as CD133, CD44, integrin α6, Sox2, Oct4, and Nanog. The anticancer effects of C9 and CsA in MKN45 GCSCs were also related to the modulation of CypA/CD147-mediated AKT and MAPK signaling pathways. These findings demonstrate the promising therapeutic potential of C9 and CsA in effectively suppressing GCSC propagation by targeting the CypA/CD147 interaction.

## Data Availability

The data that support the findings of this study are available from the corresponding author upon reasonable request.

## Supplementary Materials

The supporting information can be downloaded at: https://www.mdpi.com/article/10.3390/ijms24054734/s1.
