# Supplementary material for: Cyclophilin A Inhibitors Suppress Proliferation and Induce Apoptosis of MKN45 Gastric Cancer Stem-like Cells by Regulating CypA/CD147-Mediated Signaling Pathway
Source: Int J Mol Sci. 2023 Mar 1;24(5):4734. doi: 10.3390/ijms24054734 (PMC10003193; doi:10.3390/ijms24054734)
Supplement: Supplementary file 1 [file ijms-24-04734-s001.zip › ijms-2204440-Supplementary.pdf]

Article

Cyclophilin A Inhibitors Suppress Proliferation and Induce Apoptosis of MKN45 Gastric Cancer Stem-like Cells by Regulating CypA/CD147-Mediated Signaling Pathway

Hee Jeong Cho [1] and Hye Jin Jung [1,2,3]*

[1] Department of Life Science and Biochemical Engineering, Graduate School, Sun Moon University, Asan 31460, Korea; tjdrjdh@naver.com (H.J.C.)
[2] Department of Pharmaceutical Engineering and Biotechnology, Sun Moon University, Asan 31460, Korea
[3] Genome-Based BioIT Convergence Institute, Sun Moon University, Asan 31460, Korea
* Correspondence: poka96@sunmoon.ac.kr; Tel.: +82-41-530-2354; Fax: +82-41-530-2939

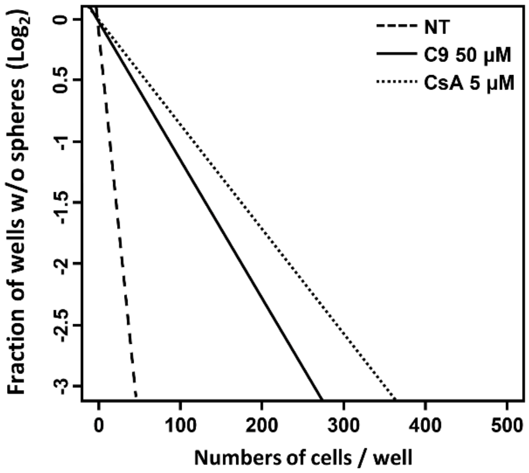

Confidence intervals for 1/(stem cell frequency)			
Group	Lower	Estimate	Upper
NT	14.9	14.7	14.5
C9 50 μM	89.4	88.2	87.0
CsA 5 μM	118.5	116.9	115.3

Figure S1. Effects of C9 and CsA on the self-renewal of MKN45 GCSCs using limiting dilution assay (LDA). MKN45-derived GCSCs were plated with limiting dilutions (from 10 to 500 cells/well) using serum-free media. The cells were then treated with C9 or CsA for 7 days and the number of wells containing spheres was quantified. The frequency of tumorsphere formation was measured by extreme limiting dilution analysis (ELDA) software (http://bioinf.wehi.edu.au/software/elda/).

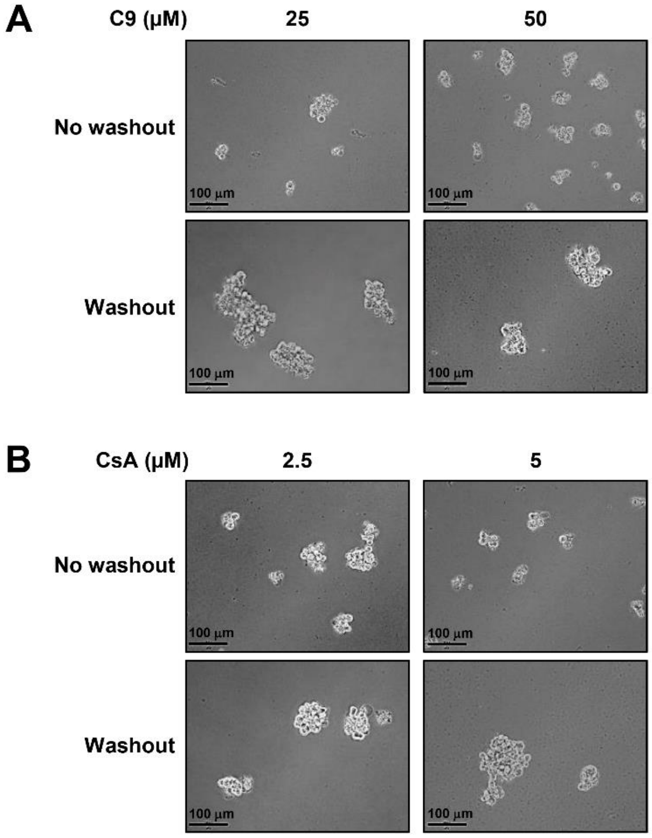

Figure S2. Reversible inhibitory effects of C9 and CsA on the growth of MKN45 GCSCs. MKN45-derived GCSCs were treated with the indicated concentrations of C9 or CsA for 24 h. The cells were washed with PBS to remove the treated compounds and then incubated in serum-free media for 6 days. Formed tumorspheres were observed under an optical microscope.

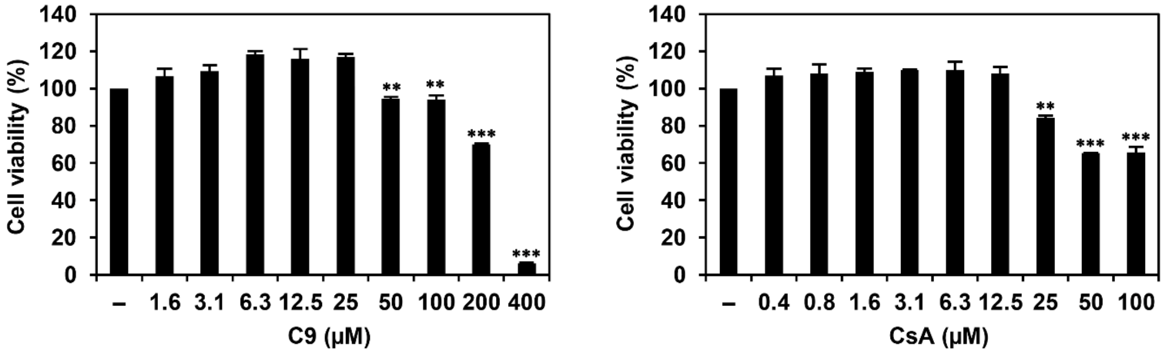

Figure S3. Effects of C9 and CsA on the viability of 267B1 human normal prostate epithelial cells. 267B1 cells were treated with the indicated concentrations of C9 or CsA for 72 h. Cell viability was measured using the CellTiter-Glo® luminescent assay system. ** $p < 0.005$, *** $p < 0.001$ vs. the control.
